# Optical and Electrical Properties of A_3_[VS_4_] (A = Na, K) Synthesized via a Straightforward and Scalable Solid-State Method

**DOI:** 10.1021/acs.inorgchem.4c00551

**Published:** 2024-05-31

**Authors:** Mohammad R. Ghazanfari, Laura Vittadello, Stephanie Bachmann, Jakob Möbs, Rüdiger Bertermann, Niklas Restel, Felix Sauerwein, Johannes C. Vrijmoed, Johanna Heine, Ann-Christin Pöppler, Mirco Imlau, Günther Thiele

**Affiliations:** † Fachbereich Biologie, Chemie, Pharmazie, Freie Universität Berlin, Berlin 14195, Germany; ‡ Department of Mathematics/Informatics/Physics, University of Osnabrück, Osnabrück 49076, Germany; § Research Center for Cellular Nanoanalytics Osnabrück, Osnabrück 49076, Germany; ∥ Institut für Organische Chemie, Universität Würzburg, Würzburg 97074, Germany; ⊥ Department of Chemistry and Material Sciences Center, Philipps-Universität Marburg, Marburg 35043, Germany; # Department of Physics, University of Oxford, Oxford OX1 3PU, United Kingdom; ∇ Institut für Anorganische Chemie, Universität Würzburg, Würzburg, 97074, Germany; ○ Fachbereich Geowissenschaften, Freie Universität Berlin, Berlin 12249, Germany; ◆ Institut für Anorganische und Analytische Chemie, Albert-Ludwigs-Universität Freiburg, Freiburg 79104, Germany

## Abstract

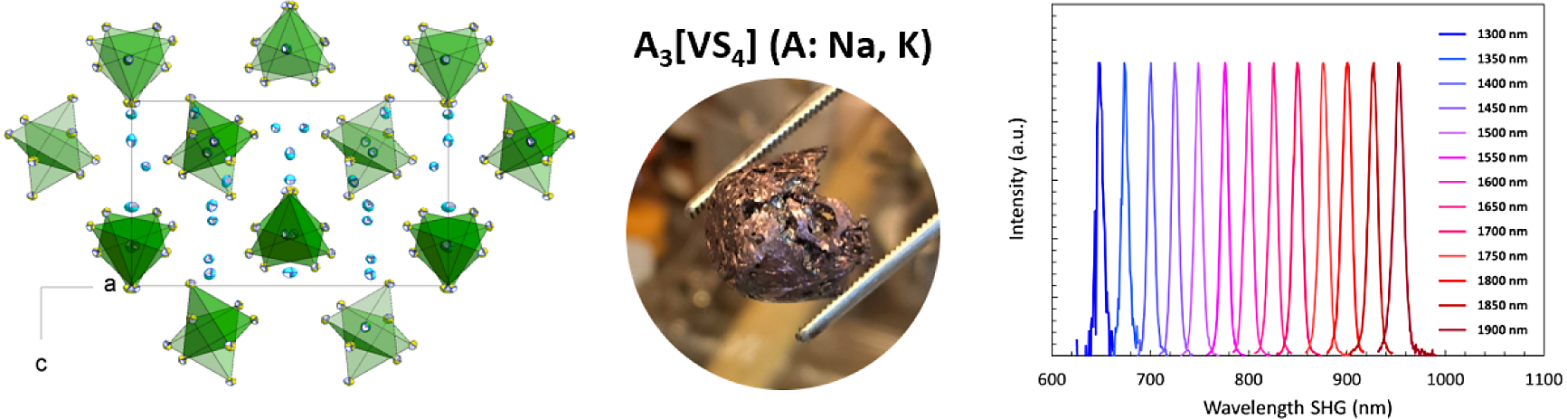

Two literature-known sulfido vanadates, Na_3_[VS_4_] and K_3_[VS_4_], were obtained through a straightforward and scalable synthetic method. Highly crystalline powders of both compounds were obtained from the homogeneous molten phases of starting materials via a—comparably rapid—solid-state technique. Low-temperature structure determination, ambient temperature powder diffraction, and solid-state NMR spectroscopy verify previous structural reports and indicate purity of the obtained samples. Both compounds show semiconductivity with the optical band gap values in the range of 2.1 to 2.3 eV. Experimental values of the ionic conductivity and dielectric constants are σ = 2.41·10^–5^ mS·cm^–1^, *k* = 76.52 and σ = 1.36·10^–4^ mS·cm^–1^, *k* = 103.67 at ambient temperature for Na_3_[VS_4_] and K_3_[VS_4_], respectively. It is demonstrated that Na_3_[VS_4_] depicts second-order nonlinear optical properties, i.e., second harmonic generation over a broad wavelength spectrum. The results introduce new aspects of sulfido vanadates as multifunctional candidates for potential optical and electrical applications.

## Abstract 2

Multifunctional A_3_[VS_4_] (A = Na, K) sulfido vanadate salts, synthesized on a gram scale, indicate semi-conductivity, high dielectric values, and intermediate ionic conduction. Na_3_[VS_4_] shows second harmonic generation in a wide range of wavelengths.

## 1 Introduction

Elemental vanadium and compounds containing vanadium have a crucial role in many current technologies, ranging from common steel productions^1^ to catalysts in chemical processes,^2^ redox flow batteries,^3^ and pharmaceutical protein inhibitors.^4^ Alkali metal vanadates are mainly associated with oxido vanadate anions such as A[VO_3_] and A_3_[VO_4_] (A = Na, K).^5−9^ There are a few literature-reported compounds containing alkali metals, vanadium, and sulfur, such as Na_*x*_VS_2_,^10^ Na_3_[VS_4_] (**1**),^11^ and K_3_[VS_4_] (**2**).^12^ Most of these compounds are only reported for their crystal structures, while physical properties are rarely studied.^12−14^ The typical synthetic approach includes conventional solid-state reactions at temperatures up to 1200 K and reaction times of several days or even weeks.^12−16^ The *ortho*-vanadates, **1** and Na_3_[VO_4_], attract much attention for potential applications as an electrode and/or electrolyte material in all-solid battery cells.^17^

Materials with noncentrosymmetric space groups can be considered for second-order nonlinear optical (NLO) phenomena, particularly for second harmonic generation (SHG). SHG refers to a second-order nonlinear optical interaction between photons and noncentrosymmetric materials such that the interaction generates photons with doubled energy of the incident photons (i.e., half of the wavelength).^18,19^ Several vanadate compounds, such as Li_3_[VO_4_],^20^ LiRb_2_[VO_4_],^20^ and Ca_3_[VO_4_]_2_,^21^ were already proven to show a NLO response, especially in the spectral range of near- and mid-infrared wavelengths for the incident light. A common feature in all of these compounds is the tetrahedral coordination of vanadium ions to create [VO_4_]^3–^ moieties. Distorted [VO_4_] moieties with a displacement of the vanadium ion from the center of the polyhedron can cause a change of the symmetry and enhance the NLO properties.^21^ Although there are several sulfido vanadates with [VS_4_]^3–^ anions, their potential as NLO materials is still unknown.

**1** was synthesized for the first time in 1997 by Klapp and Gabl as an analogue to the Na_3_[PO_4_] structure by heating a mixture of binary and elemental starting materials at 1073 K for 5 days, according to eq 1.^11^
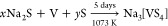
1

**Figure 1:**
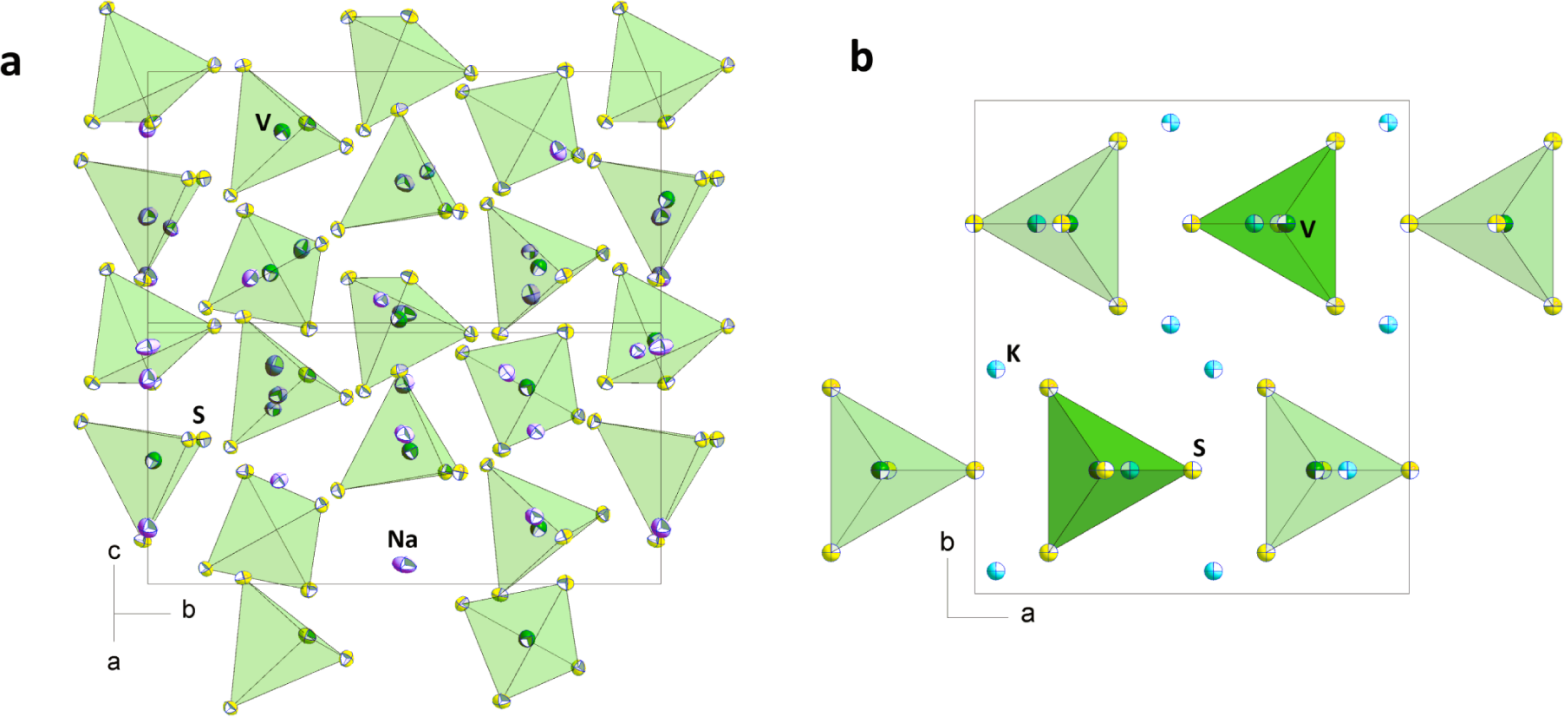
(a) Excerpt of the crystal structure of **1**, with coordination polyhedra of isolated [VS_4_]^3–^ tetrahedra. (b) Excerpt of the crystal structure of **2**, with coordination polyhedra of isolated [VS_4_]^3–^ tetrahedra. Selected bond lengths and angles reported at room temperature in **1**: V–S: 2.134(8)–2.163(3) Å, Na–S: 2.830(4)–2.992(11) Å, S–V–S: 108.816(41)–110.792(31)°, and in **2**: V–S: 2.147(6)–2.163(3) Å; K–S: 3.181(8)–3.420(9) Å; S–V–S: 108.792(51)–109.245(51)°.

The authors only reported the crystal structure of **1** without any description on the employed ratio of the starting materials, nor was the yield or phase purity reported. **1** crystallizes in the space group *P*2_1_*c* (114) with the vanadium ions tetrahedrally coordinated by sulfur ions to give eight isolated [VS_4_]^3–^ anionic moieties per unit cell. According to the literature, the cell parameters are as follows: *a* = 13.502(1) Å and *c* = 7.9504(8) Å at room temperature.^11^**1** has the crucial prerequisite of a noncentrosymmetric space group for potential second-order NLO applications. Figure 1a displays the crystal structure of **1**. In 2019, He et al. synthesized **1** by heating the identical starting materials as in eq 1 to 773 K for several hours and studied its sodium ionic conductivity in comparison to the phosphorus doped compound.^22^ However, they reported small amounts of impurities of V_2_O_5_ (2.6 wt %) and elemental sulfur (6.7 wt %) as side products. The effects of the crystal structure on the ionic conductivity (1.16·10^–5^ mS·cm^–1^ at 298 K) of **1** was studied based on density functional theory computations.^23^

**2** was introduced by Dürichen and Bensch in 1996, synthesized through a high temperature reaction at around 1073 K using a molten flux method.^12^**2** crystallizes in the space group *Pnma* (62) with *a =* 9.138(2) Å, *b* = 10.627(2) Å, and *c* = 9.131(2) Å.^12^ Although the crystal structure is described, there is no report on its physical properties. The crystal structure of **2** is depicted in Figure 1b.

Based on our previous works on (*pseudo-*)tetrahedral metalates with sulfide ligands,^24^ we were interested in extending our synthesis strategy and subsequent analysis to **1** and **2** to investigate dielectric, electrochemical impedance, optical, and NLO properties.

## 2 Experimental Section

### 2.1 Materials and General Procedure

As majority of the starting materials and products are air- and moisture-sensitive, all manipulations including the preparation of the starting materials, the synthesis, and the characterization of the products were conducted under argon using a glovebox and/or standard Schlenk techniques.

Sodium (*Onyxmet*, 99.8%), potassium (*Acros Organics*, 98%), sulfur (*abcr*, 99% sublimed), vanadium (*Onyxmet*, 99.9%), and naphthalene (*Acros Organics*, 99%) were purchased and employed without any further purification. Tetrahydrofuran (THF) was dried using sodium as a drying agent by refluxing for 8 h at around 339 K, while pyridine was dried similarly using a drying agent of CaH_2_ at around 388 K. Na_2_S and K_2_S were synthesized in THF and liquid ammonia, respectively, according to the literature-reported methods.^25,26^ Liquid ammonia was condensed from the ammonia gas (*Linde*, 99.99%). For the synthesis of Na_2_S, 7.53 g (0.23 mol, 1 equiv) of sulfur and approximately 30 mg of naphthalene were placed in a Schlenk flask including 100 mL of THF. 10.82 g (0.47 mol, 2 equiv) of sodium was added to the flask and the mixture was stirred for around 4 h. The off-white precipitate of Na_2_S is washed with THF multiple times and dried under vacuum. To synthesize K_2_S, 20.26 g of potassium (2 equiv, 0.513 mol) was dissolved in liquified ammonia at about 200 K, then 8.30 g of sulfur (1 equiv, 0.257 mol) was gradually added to the solution at a temperature of 240 K and stirred for a few hours. The ammonia is slowly evaporated by allowing the reaction setup to warm to ambient temperature. Around 3 wt % of ammonia remains trapped in the product powder. The colors of as-synthesized Na_2_S and K_2_S were off-white and light yellow, respectively. The purity of Na_2_S and K_2_S was evaluated and verified by powder X-ray diffraction (PXRD). Extreme care has to be taken when working with liquified ammonia, and pressure release options have to be connected throughout the synthesis. Potassium–ammonia solutions, as well as finely ground K_2_S (especially in case of a surplus of potassium) can be pyrophoric. Corresponding safety precautions must be considered.

#### Synthetic Procedure of **1**

4.7061 g (0.0603 mol, 1.5 equiv) of Na_2_S, 2.0478 g of vanadium (0.0402 mol, 1 equiv), and 3.222 g (0.1005 mol, 2.5 equiv) of sulfur were mixed thoroughly, placed into a silica fuse ampule, and heated to around 1173 K for around 5 min using an oxygen–methane flame torch to make a homogeneous molten phase.

After the ampule has cooled to ambient temperature, it is carefully broken and the product is collected and grounded. The reaction yields 9.3 g (93%) of **1** in the form of dark purple powder.

#### Synthetic Procedure of **2**

In a similar procedure, **2** was synthesized by mixing 5.735 g (0.052 mol, 1.55 equiv) of K_2_S, 1.7167 g vanadium (0.0337 mol, 1 equiv), and 2.6998 g (0.0842 mol, 2.5 equiv) of sulfur and heated to around 1273 for 10 min. A slight excess of K_2_S was added to compensate for the weight of unevaporated ammonia (trapped in the powder). The reaction yields 9.5 g (95%) of **2** in the form of dark brown powder.

#### Single Crystals Preparation Procedure

For both **1** and **2**, the crystallinity and size can be enhanced through a solvothermal treatment. Around 0.125 g of as-synthesized powder was placed in a 10 mL pressure glass vial (with caps ensuring a controlled pressure release above 3 bar) with 2 mL of pyridine and heat treated at 423 K for 48 h (using a dry block thermostat) to yield plate-shaped crystals of dark violet color for **1**, and block-shaped crystals of brown color for **2**. Details about the obtained crystals including dimensions and structural parameters are available in the Electronic Supporting Information (ESI).

### 2.2 Structural and Microstructural Analyses

#### Single Crystal X-Ray Diffraction (SC-XRD)

Single crystals of **1** and **2** were isolated and collected under an optical microscope, mounted in Paraton oil, and analyzed using a *Bruker D8 Venture* diffractometer with Mo–K_α_ radiation (λ = 0.71073 Å) at 130 K. The crystal structures were processed and refined in Olex 2^27^ using *ShelXT*(28) and *ShelXL*(29) programs. The crystal structure depictions were prepared using *DIAMOND4.5.2* software package.^30^

#### Powder X-Ray Diffraction (PXRD) and Rietveld Structure Refinement

PXRD samples were prepared by placing approximately 30 to 50 mg powder on a self-printed sample-holders based on the reported protocol,^31^ on a *Malvern Panalytical Empyrean* using Cu–K_α_ radiation (λ = 1.54184 Å). The PXRD measurements were conducted at 293 K in the 2θ range of 10° to 90°. The structural details of **1** and **2** such as crystallinity and crystallite size were extracted by Rietveld structure refinement^32^ using *GSAS II*(33) software. More details on the Rietveld refinement are provided in the ESI.

#### Energy-Dispersive X-Ray Spectroscopy

Around 50 mg of fine powder was deposited on a measurement stub and placed into a scanning electron microscope (SEM, *Zeiss Sigma 300VP* field-emission scanning electron microscope) coupled with an energy-dispersive X-ray spectroscopy (EDX, *Bruker, Quantax Xflash 6,* 60 mm^2^ SSD EDS) detector. Measurements were conducted at a working distance of 8.7 mm and a beam energy of 20 kV. The postprocess quantitative analysis of the EDX measurements was carried out by using the *Bruker Esprit 2.1* software.

#### Thermal Analyses

Thermal properties of **1** and **2** were evaluated based on the thermal gravimetry (TG) coupled with differential scanning calorimetry (DSC) measurements conducted under nitrogen flow in the temperature range of 300 to 773 K by pacing the fine powders in alumina crucibles and measured using a *STA 449 F3 Jupiter* thermal analyzer.

#### Nuclear Magnetic Resonance (NMR) Spectroscopy

The samples were measured with a *Bruker Avance Neo spectrometer* at 9.4 T with a spinning rate of 10 kHz for **1** and 12 kHz for **2**. A rotor diameter of 4 mm and a *HX* probe was used. Sample preparation was done in an argon-filled glovebox. Although the measurements were performed at room temperature, the sample temperature during the measurement is higher due to frictional heating during magic angle spinning (MAS). The pulse length for ^23^Na and ^51^V one-pulse experiments was 4.25 and 3 μs, respectively. As the vanadium signals have a wide chemical shift range, the transmitter frequency offset was shifted in intervals of 800 ppm to cover the whole signal range and the spectra were reassembled after the measurements. Different spinning rates were used to identify the isotropic signals. All chemical shifts were calibrated by setting the ^13^C low-field signal of adamantane to δ = 38.48 ppm by adjusting the field value of the spectrometer according to the IUPAC recommendations,^34^ with Ξ[^13^C] = 25.145020 MHz, Ξ[^23^Na] = 26.451900 MHz, and Ξ[^51^V] = 26.302948 MHz.

For the fitting of the signal pattern, the *Solid Lineshape Analysis (sola) module (version 2.2.4 (2013)) of Bruker TopSpin 4.3* software was used. Here, beside the chemical shift value and the signal broadening, the parameters *C*_*Q*_ (quadrupolar coupling constant) and *η*_*Q*_ (quadrupolar asymmetry parameter) can be adjusted to fit the signals characterizing the properties of the quadrupolar ^51^V nucleus. The values of *C*_*Q*_ and *η*_*Q*_ were found to be similar for all signals. Only the first four spinning side bands were considered, as the excitation bandwidth affects signal intensity.

### 2.3 Dielectric and Impedance Measurements

#### Preparation of Pellets and Sintering

To prepare the bulk samples, approximately 400 mg of fine powder was placed into the cylindrical pressing mold and uniaxial pressure up to 80 kN was applied using a uniaxial hydraulic press (*Paul Weber Maschinen*, 200 kN). The pressed pellets were transferred to alumina crucibles and sintered at 673 K for 12 h. The sintered pellets had a shining dark brown color (for both **1** and **2**) with dimensions of 12.9 and 13 mm in diameter and 2.1 and 2.4 mm in thickness for **1** and **2**, respectively. To conduct dielectric and electrochemical impedance measurements, the pellets were placed into a self-made airtight sample holder (see ESI) between two pellets of graphite as current collectors. The graphite pellets were pressed using a similar procedure and employed without any sintering. Dimensions of graphite pellets are 13 mm in diameter and 1–1.5 mm in thickness. Dielectric measurements including dielectric constant (calculated based on the measured capacitance) and dielectric loss were carried out at ambient temperature using a precision LCR meter (*SOURCETRONIC*, ST2829C) in the frequency range of 0.02 to 1000 kHz with the applying voltage of 1 V without any additional bias voltage. Complex impedance measurements of samples were conducted at ambient temperature using an electrochemical impedance analyzer (EIS, *BioLogic* MTZ-35) in the frequency range of 100 mHz to 1 MHz. The applied sine phase of the measurements was 100 mV with a resolution of 50 μV. Calculations of the ionic conductivity values were based on the Nyquist equation.^35^ The simulated plots were calculated according to the designed equivalent circuit using *ZSimpWin* program.^36^

### 2.4 Optical Properties

#### NLO Measurements

Approximately 300 mg of fine powder of **1** was pressed as a powder pellet using a uniaxial hydraulic press according to the approach explained above, transferred to a self 3D-printed sample holder,^19^ and sealed using standard electrical tapes to prevent it from air exposure. The holder had two fused silica glass plates on both sides as a transparent window for the laser beam. The second-order nonlinear optical properties of the sample were analyzed via the diffuse femtosecond pulse reflectometry technique.^37^ Briefly, the laser source, composed of a regeneratively amplified femtosecond pulse laser (Pharos-HE-20, *Light Conversion Inc.*) and an optical parametric amplifier (*Orpheus F, Light Conversion Inc.*, OPA) with a pulse duration of about 170 fs, was focused onto the pellet surface via an off axis parabolic mirror (MPD169-P01) at a normal incidence.^37^ The diffusive emitted light in the visible was detected in reflection geometry by using a fiber spectrometer (QEPro, *Ocean optics Inc.*). A long pass filter (FEL1150, *Thorlabs*) was used to suppress the residual OPA light emission. Power-dependent measurements were performed at a fixed fundamental wavelength of 1400 nm with a repetition rate of 10 kHz in the power range 20–57 mW and an integration time of the detector of 1 s. The power was adjusted by means of a continuous variable neutral density filter wheel (NDC-25C-2, *Thorlabs*) and for every value of the power at least three measurements were averaged. Wavelength-dependent measurements were conducted by tuning the OPA in the range from 1300 up to 1900 nm (repetition rate: 5 kHz, power: ∼ 11 mW) and integration time of 2 s. The energy density threshold of the SHG was calculated according to the relation *E* = P·*t·A*, where *P* is the average power, *t* the integration time of the spectrometer, and *A* the illuminated area.

#### UV–Visible Spectroscopy

Optical absorption spectra were recorded on a Varian Cary 5000 UV/vis/NIR spectrometer in the range 200–800 nm in diffuse reflectance mode by employing a Praying Mantis accessory (Harrick) with automatic baseline correction. BaSO_4_ was used as white standard, and the compounds were ground together with BaSO_4_ (5% of the compound by weight) prior to the measurement to dilute the sample and reduce artifacts.

## 3 Results and Discussion

### 3.1 Synthesis and Structural Properties

**1** and **2** were successfully synthesized through a straightforward solid-state reaction according to the reaction equations 2 and 3, respectively.
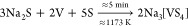
2
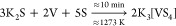
3

**Figure 2:**
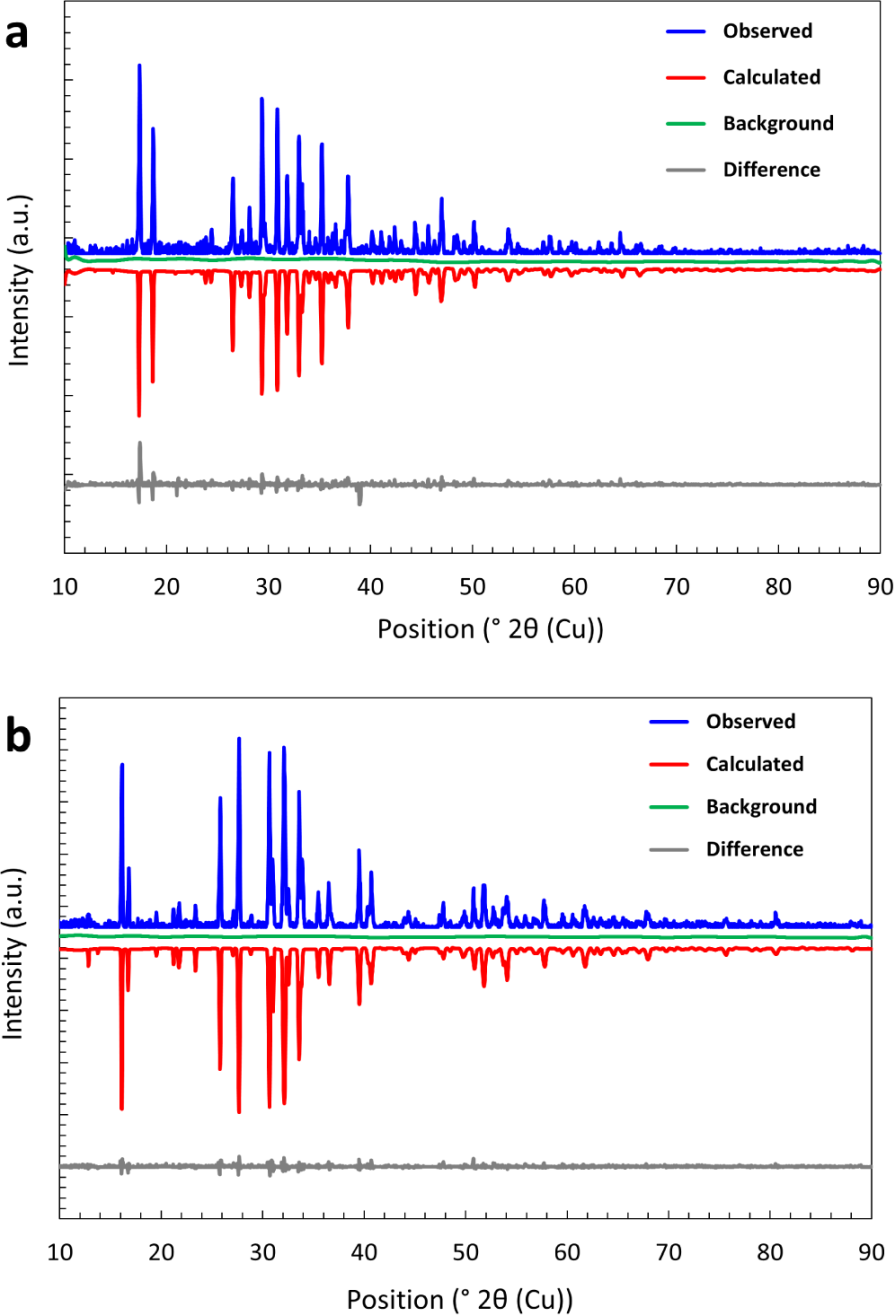
(a) Rietveld refinement results of the as-synthesized **1**. (b) Rietveld refinement results of as-synthesized **2**.

The yields of the synthetic reactions were 93% and 95% for **1** and **2**, respectively, while the reactions are scalable up to 50 g per batch, only limited by the size of the reaction containers. Minor yield loss (around 7 and 5% for **1** and **2**, respectively) results from residual material at the reaction container walls. According to single crystal XRD measurements, **1** crystallizes in the space group *P*2_1_*c* (114), as already reported in the literature. However, the cell parameters, *a* = *b* = 13.4403(6) Å, *c* = 7.9073(5) Å) are slightly shorter than the reported values (*a* = *b* = 13.502(1) Å and *c* = 7.9504(8) Å) due to the different measurement temperatures, 130 K in this work compared to 293 K in the literature.^11^ In a similar way, **2** crystallizes in the already reported space group *Pnma* (62), with the comparably smaller cell dimensions *a* = 9.0902(12) Å, *b =* 10.4946(12) Å, and *c* = 9.0832(11) Å at 130 K compared to *a* = 9.138(2) Å, *b =* 10.627(2) Å, and *c* = 9.131(2) Å at 298 K.^12^ The phase purity of **1** and **2** was investigated and verified by Rietveld structure refinement as well as EDX analyses (Figure S1). Figure 2a,b indicates the Rietveld refinement pattern of **1** and **2**, respectively. According to the refinements, both **1** and **2** are pure with a degree of crystallinity of 95.8 and 97.0% ± 3%, respectively. The elemental composition of both compounds was additionally evaluated using EDX analysis, and the stoichiometric ratio of 3:1:4 is verified for Na:V:S in **1** and K:V:S in **2**, confirming the purity of the compounds. Further details of the crystal structure and powder refinements, structural parameters as well as EDX measurements and results are provided in the ESI.

**Figure 3:**
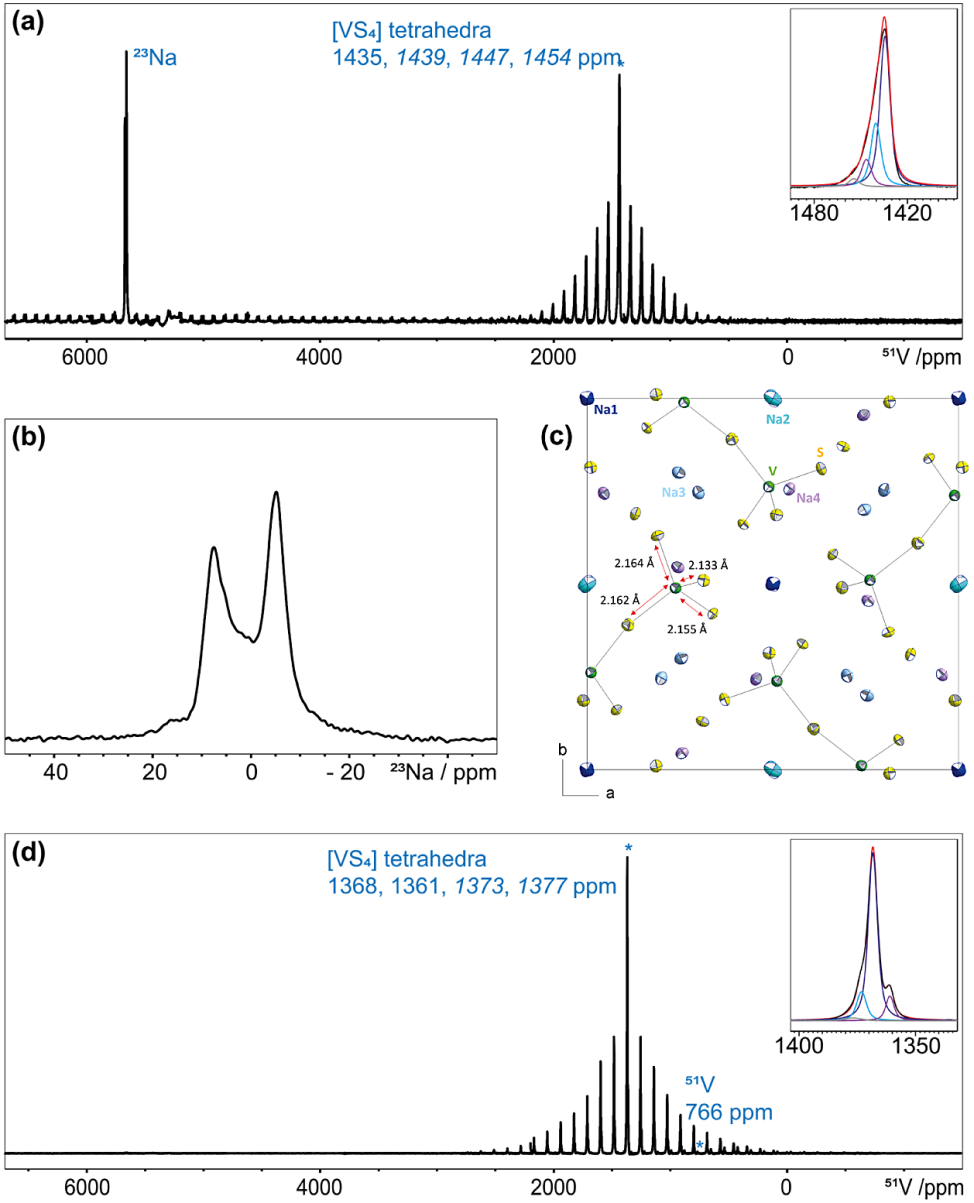
(a) ^51^V direct polarization (DP) MAS of **1** at 10 kHz (chemical shift range: + 6800 to −1600 ppm, measured as variable offset spectra) recorded with 32 scans and a recycle delay of 60 s. (Inset) Close-up view of fitting of the isotropic chemical shift at 1435 ppm and nearby signals (b)^23^ Na DP MAS spectra of **1** measured at 10 kHz with 32 scans and a recycle delay of 10 s. (c) Excerpt of the crystal structure depiction of **1**, showing the individual sodium ion types as well as distorted [VS_4_] units. (d) ^51^V DP MAS of compound **2** measured at 12 kHz (chemical shift range: + 7000 to −2400 ppm, measured as variable offset spectra) and with 16 scans and a recycle delay of 60 s. (Inset) Close-up view of the fitting result of the isotropic chemical shift at 1368 ppm and nearby signals.

To further assess the purity and obtain chemical shift values, solid-state NMR spectroscopic measurements of ^23^Na and ^51^V for **1** and ^51^V for **2** were performed. The spectra were recorded with different spinning speeds to identify the isotropic chemical shifts (Figure S2). ^23^Na (*I* = 3/2) and ^51^V (*I* = 7/2) are quadrupolar nuclei. This means that the charge in the nucleus is not distributed spherically, and therefore, the line shape is dominated by quadrupolar interactions and must be simulated to extract the isotropic chemical shift. This effect is not observed in the case of an ideal cubic symmetry of the respective nucleus coordinated either in a tetrahedral (*T*_*d*_) or octahedral (*O*_*h*_) arrangement. Figure 3a displays the ^51^V NMR spectra of **1** with the main signal at 1435 ppm. This signal can be assigned to the tetrahedral [VS_4_] units.^38^ Additionally, a shoulder toward higher ppm values is observable, which can be explained by a slight deviation from the cubic symmetry. This deviation agrees with the reported distortion in the [VS_4_] units in Na_3_[VS_4_].^22^ In contrast to the assumption made in literature,^38,39^ chemical shifts δ(^51^V) > 5000 ppm are assigned to^23^ Na. As Lamor frequencies are similar, signals of both nuclei might also be detected during this measurement. Figure 3b shows the^23^ Na NMR spectra of **1** including three signals at 7.6, 2.8, and −5.1 ppm attributed to the crystallographically independent sodium ions. The respective coordination polyhedra as well as the distorted [VS_4_] units are shown in Figure 3c. In the [VS_4_] units, V–S bond lengths indicate small differences, resulting in slight distortions of the tetrahedra, which give rise to first-order quadrupolar interaction (Figure S3). In addition, it is worth mentioning that the presence of a nonzero asymmetric parameter *η*_*Q*_ points toward the presence of second-order nonlinear optical effects.^40−42^

In the ^51^V NMR spectra of **2** (Figure 3d), there is a main signal at 1368 ppm and three further signals at 1361, 1373, and 1377 ppm, which can be assigned to slightly different [VS_4_] units with different amounts in the powder. The signal pattern can be explained by four sites and the corresponding first-order quadrupolar coupling (Figure S3). Table 1 lists the evaluated chemical shifts and the fitting parameters (Goodness of Fit: 94.7%) for signals observed in the NMR spectra of **2**.

**Table 1:** Fitted ^51^V Chemical Shifts and their Parameters for Signals Observed in the NMR Spectra of **2**

	site 1	site 2	site 3	site 4
Proportion (%)	76	10	12	2
Chemical shift (ppm)	1368	1361	1373	1377
*C*_*Q*_ (kHz)	653	650	720	792
*η*_*Q*_ (0–1)	0.546	0.441	0.77	0.6

The obtained values characterize the chemical environment around the vanadium atoms. Especially, sites 3 and 4 are broad and only recognizable as a shoulder. The peak shape and the small range of the chemical shifts (on a scale of vanadium signals) lead to the conclusion that the [VS_4_] tetrahedra are present in the structure. However, there might also be small disorders/distortions in the lattice of the cations and long-range interactions resulting in the observed *C*_*Q*_ and *η*_*Q*_ values.

There is an additional signal at 766 ppm, which is already reported without assignment for a [VS_4_] containing compound, Mg_3_V_2_S_8_.^38^ This signal might be attributed to the mixed oxido-sulfido vanadate [VOS_3_]^3–^ (740 ppm) and/or H[VOS_3_]^2–^ (748 ppm), as reported in solution NMR studies.^43^ Such impurities, which are not detectable by PXRD, might be obtained by partial oxidation of the compounds during synthesis, sample transfer, or handling or during the measurements. We note that substitution of sulfur and oxygen ions from the fused silica glass during the high temperature synthesis might be another source of these trace impurities.

The thermal profiles of **1** and **2**, studied using a thermogravimetric analysis coupled with differential scanning calorimetry (TG/DSC), provide no event during heating and cooling cycles in the temperature range of 25 to 773 K, indicating thermal stability of both compounds up to 773 K.

### 3.2 Electrical Properties

**Figure 4:**
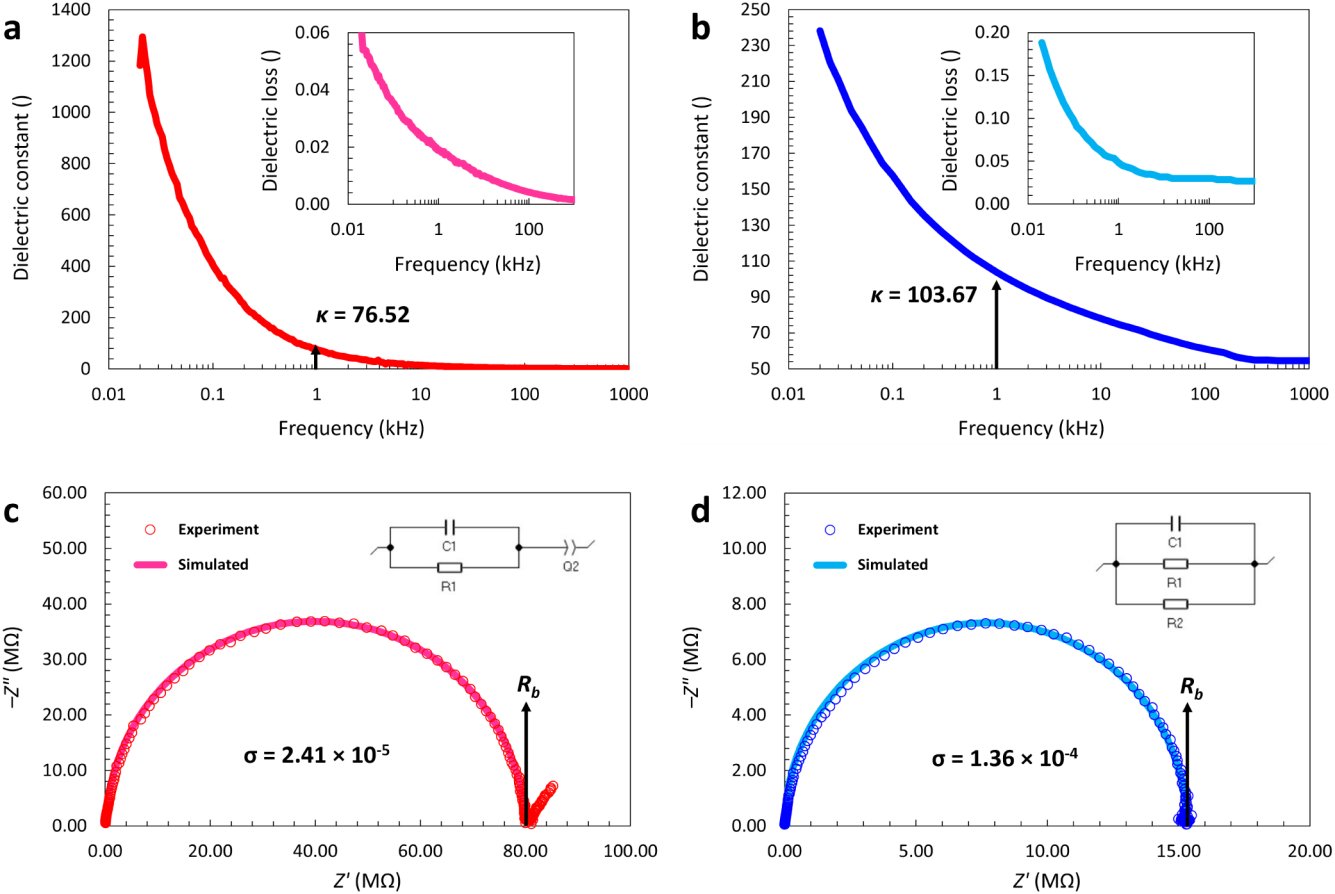
(a) Plot of the dielectric constant of **1** as a function of the measurement frequency. Inset: Plot of the dielectric loss of **1** as a function of measurement frequency. (b) Plot of the dielectric constant of **2** as a function of measurement frequency. Inset: Plot of the dielectric loss of **2** as a function of the measurement frequency. (c) Complex impedance plot (imaginary part of impedance, *Z”*, as a function of real part of impedance, *Z’*) of **1** and the simulated curves according to an equivalent circuit (inset) including a capacitor (C1) and a resistor (R1) in parallel, with a constant phase element (Q2) in series. (d) Complex impedance plot (imaginary part of impedance, *Z”*, as a function of real part of impedance, *Z’*) of **2** and the simulated curves according to an equivalent circuit (inset) including a parallel set of a capacitor (C1), a resistor for the ionic characteristic (R1), and a resistor for the electronic leakage (R2).

Dielectric and electrochemical impedance were measured for pellets of **1** and **2**, sintered at 673 K. Dielectric constant values were calculated based on the following equation:^44^
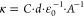
4

where *κ* is the dielectric constant, *C* is the experimental electrical capacitance, *d* is the thickness of the pellet, ε_0_ is the electrical permittivity of vacuum space equaling 8.85 · 10^–12^ m^–3^ kg^–1^ s^4^ A^2^, and *A* is the contact surface area between the pellet and electrodes. Figure 4a,b illustrates the variation of the dielectric constants of **1** and **2** as a function of the measurement frequency. For both compounds, the dielectric constant has comparably high values at low frequencies. Increasing the frequency, the *κ* values sharply decrease and then continue to gradually decrease to a plateau trend. The sharp decrease can be explained by a deactivation of the space charge polarization mechanism, as a dominant polarization mechanism at low frequencies. The decreasing rate of the dielectric constants is much sharper for **1** with a decrease from around 1300 at low frequencies of around 20 Hz to around 76 at 1 kHz. The dielectric constant values of **1** and **2** are 76.52 and 103.67, respectively, at room temperature and a frequency of 1 kHz as a common standard working frequency. These values are higher than that of the common reference material (SiO_2_, around 3.9 at 1 kHz) for potential applications in the semiconductor manufacturing such as gate dielectric materials and transistors.^45^ The variations of the dielectric loss values of **1** and **2** as functions of the frequency increase are depicted in the inset frames of Figure 4a,b, respectively. Similar to the dielectric constant, dielectric loss values are sharply decreased at initial frequencies (0.2 to 1 kHz) and then slowly reduced to a plateau level. In the whole frequency range of 0.02 to 1000 kHz, the dielectric loss values are lower than 0.1, while the loss values at 1 kHz are 0.0188 for **1** and 0.483 for **2**. There is no report in the literature for the dielectric properties of the alkali metal oxido and sulfido vanadates. However, the experimentally measured values of **1** and **2** are in the similar range or comparably higher than the reported values for a few other vanadate compounds such as Bi[VO_4_] (κ ≈ 68),^46,47^ Zr[V_2_O_7_] (κ ≈ 62),^48^ Ba_2_[V_2_O_7_] (κ ≈ 9.5), and BaZn[V_2_O_7_] (κ ≈ 10).^49^

Figure 4c,d displays the complex electrochemical impedance plots (the imaginary part of impedance, *Z”*, as a function of the real part of impedance, *Z’*) of **1** and **2**, respectively. Both plots provide semicircular arcs, and results for **1** show an additional tail in the low frequency range. The semicircular shape of the curve and the ending tails indicate a purely ionic characteristic of the electrical conductivity in **1**. Although the complex impedance plot of **2** displays a semicircular arc, there is no obvious tail at low frequencies, suggesting a contribution of the electronic conductivity to the total electrical conductivity. The ionic conductivity values are calculated based on the Nyquist equation as follows:^35^
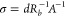
5

where *σ* is the ionic conductivity, *d* is the thickness of the pellets, *A* is the surface area of the electrode, and *R*_*b*_ is the bulk resistivity which is obtained according to the intercept of the semicircular edge of the complex impedance curve with the real impedance (*Z’*) axis. At ambient temperature, the ionic conductivity values of **1** and **2** are 2.41·10^–5^ mS·cm^–1^ and 1.36·10^–4^ mS·cm^–1^, respectively. For both **1** and **2**, the complex impedance plots are simulated based on the equivalent electrical circuits. For **1**, the best fitting of simulated and experimental results corresponds to an equivalent circuit of a resistor (R) and a capacitor (C) in parallel, representing the capacitance and resistance behaviors of the bulk sample, in series with a constant phase element, CPE, (Q), accounting for interfacial effects of the electrodes (ending tail part on the plot).^50^ For **2**, the equivalent circuit includes a capacitor and a resistor for the ionic conductivity in addition to another resistor element due to the potential electronic leakage, all in parallel. The measured value for **1** is in a good agreement with the reported value (σ = 1.16·10^–5^ mS·cm^–1^ at 298 K).^22^ The small difference of the measured value and the reported value could be explained by different sample preparation procedures. Here, the pellets were sintered at 673 K, while the samples in the literature were not reportedly sintered. The sintering process can enhance the bulk density of the samples, thereby increasing the ionic conductivity.^51^ A different measurement temperature or other measurement parameters, such as the applied voltage, not reported in the literature, might be additional reasons for the observed difference in conductivity values, as well as the reported impurities of previous samples. While some compounds containing alkali metals, oxygen, and vanadium, such as Na_3_[VO_4_],^17^ NaVO_3_,^52^ and K_2_[V_3_O_8_],^53^ are recently reported as promising candidates for the electrode materials of the alkali metal ion batteries (σ ≈ 0.1–10 mS·cm^–1^), **1** and **2** display ionic conductivity values comparable with some vanadium-containing materials such as ZrV_2_O_5_ and Ag_3_Sc_2_[VO_4_]_3_.^54^

Although the ionic conductivity of **1** is not in the range of superionic conducting materials for use in Na-ion battery cells, it is still in an intermediate range of the reported sodium ionic conductors. For **2**, to the best of our knowledge, no complex impedance and ionic conductivity values are reported. The ionic conductivity value of **2** is well below the range of highest potassium-ion conductors (1 to 35 mS·cm^–1^).^55^

### 3.3 Optical Properties

**Figure 5:**
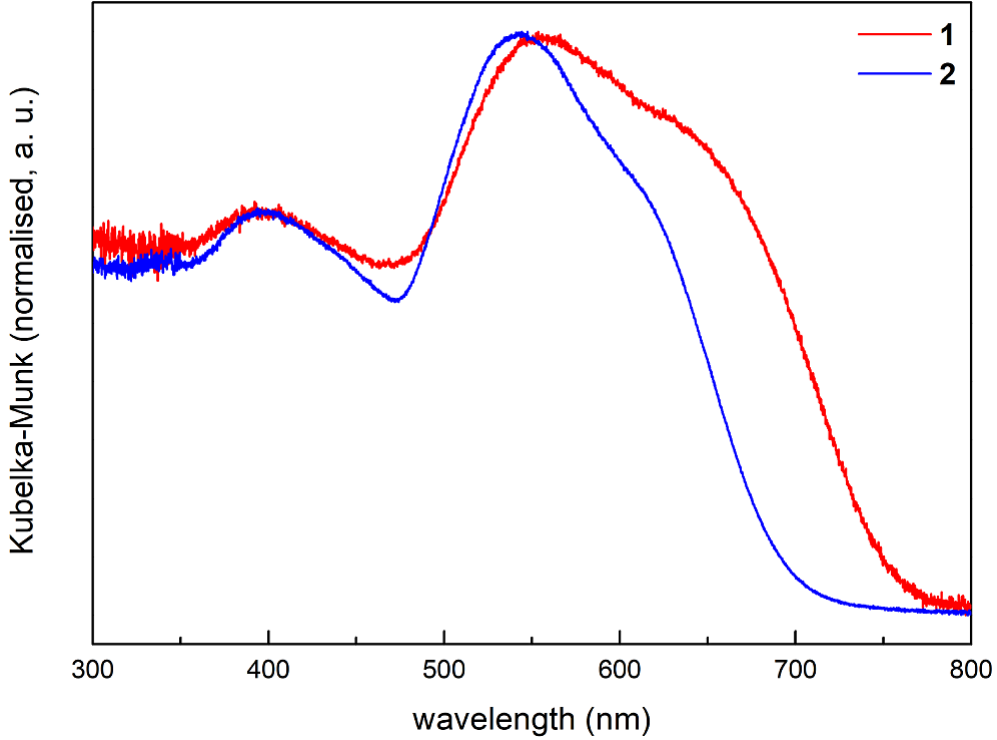
UV–visible spectra of **1** and **2.** Absorption data were calculated from diffuse reflectance measurements.

The optical properties of **1** and **2** were studied by UV–visible spectroscopy. Figure 5 shows the spectra, which, for ease of viewing, are converted from diffuse reflection to absorption using the Kubelka–Munk function. Both spectra show complex patterns of overlapping absorption bands superimposed on what can from its shape be described as the absorption edge of the band gap. Therefore, it is only possible to roughly estimate the band gap energy from the spectra. When assuming a direct nature of the transition, analysis of the Tauc plots yields gap energies of 2.1 and 2.3 eV for **1** and **2**, respectively (see Supporting Information for details). The overall onset of absorption, including the bands preceding the band edge, is at 754 nm (1.64 eV) for **1** and 692 nm (1.79 eV) for **2** and the absorption maxima lie at 556 nm (2.23 eV) and 543 nm (2.28 eV), respectively. This is very close to what has previously been found by Röhr and coworkers, who reported an absorption maximum of 560 nm (2.2 eV) for both compounds.^56,57^ However, due to a different measurement setup (transmission of pressed KBr pellet), the feature is much less distinct in their spectra. In addition, Röhr and coworkers performed an in-depth computational investigation both on the isolated [VS_4_]^3–^-anions and the extended solids and found that the main transition around 560 nm as well as the second, less intense one around 400 nm can be assigned to pure S-*p* to V-*d* charge transfer excitations, giving raise to additional electronic states within the band gap.^56,57^

**Figure 6:**
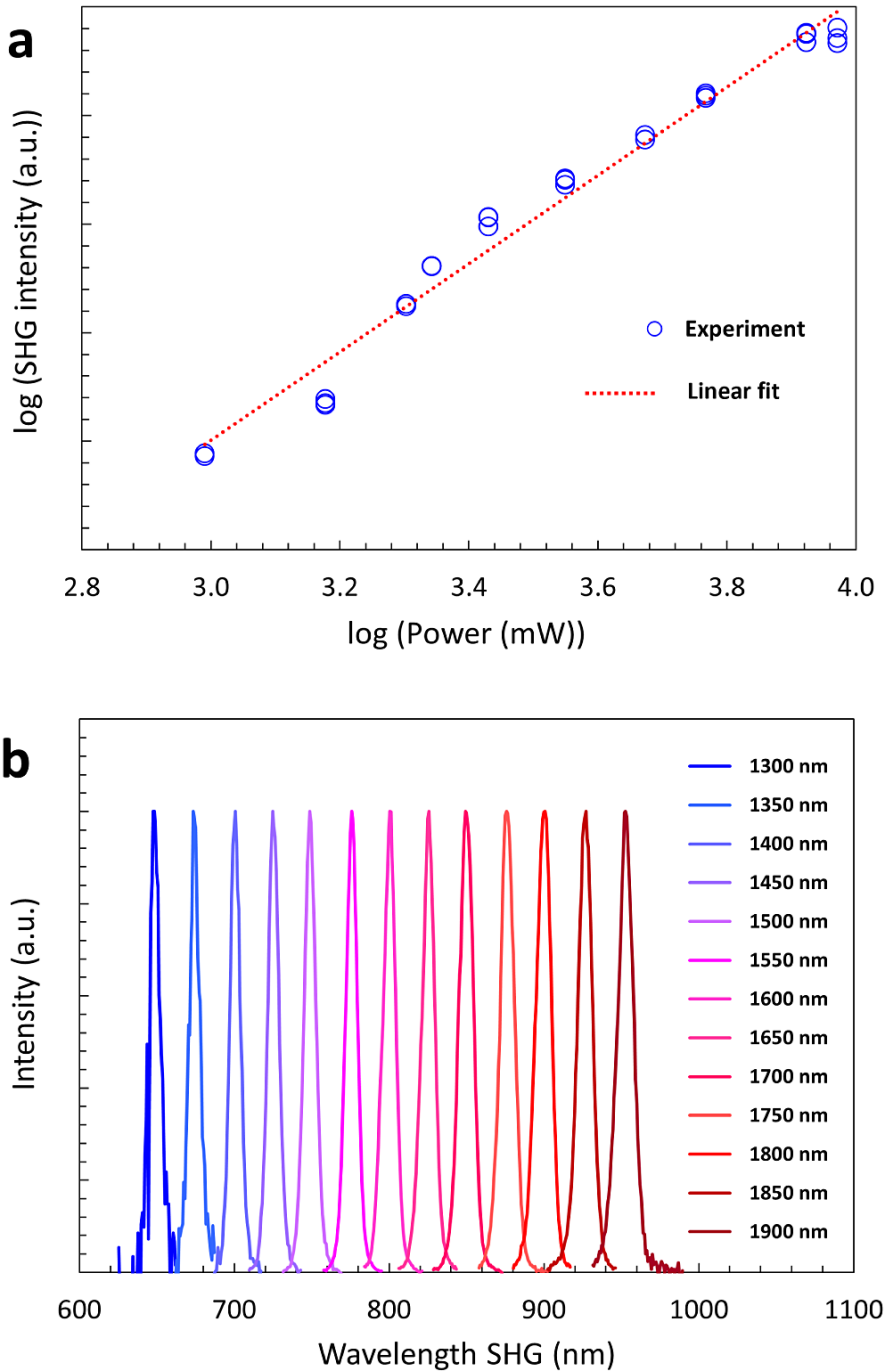
(a) Variation of SHG intensity of **1** as a function of incident laser power, indicating a quadratic relationship with the linear fit slope of 2.0 ± 0.1. (b) Normalized SHG behaviors of **1** as a function of different wavelengths of the incident beams, in the range of 1300 to 1900 nm, displaying the arbitrary SHG in the whole wavelength range.

The noncentrosymmetric space group of **1** warrants the determination of NLO properties, here based on the study of second harmonic generation (SHG). To verify the SHG nature of the emitted light, the intensity dependence as a function of the average power of the laser is investigated. The theory prescribes a quadratic relationship between these two quantities, when the other laser parameters like repetition rate, pulse duration, and area of the fundamental beam remain unchanged, and other NLO processes are neglected. Figure 6a shows the result of this study, where the intensity change of the generated diffuse reflected SHG is plotted as a function of the average power of the incident beam. Given the double logarithmic scale, the slope of the linear fit of 2.0 ± 0.1 indicates the expected quadratic relationship. This result, combined with the fact that the light is emitted at half of the pumping wavelength, as it is validated by analyzing the spectra, indicates that the light conversion mechanism is based on SHG, in contrast to other types of NLO phenomena. A similar result is obtained by applying an incident beam with the wavelength of 1300 nm, where the slope is determined to be 2.1 ± 0.2. The laser-induced damage threshold for the observed SHG behavior at 1400 nm of **1** is of 3·10^5^ W·m^–2^. To validate the possibility to continuously generate SHG over a large spectral range, the fundamental laser wavelength is changed in the range of 1300 to 1900 nm with a step-size of 50 nm. Figure 6b displays the obtained intensity normalized SHG emission spectra. As a result, the SHG can be generated at arbitrary wavelengths in the studied spectral range. The possibility to create harmonic emission in such a continuous way without satisfying the phase matching condition indicates that the dimension of the sample, in particular of the crystallite average size, is below or in the range of the coherence length of the harmonic process.^19^ The latter can be assumed in the range of tens of micrometers, considering that the material analyzed here has a refractive index similar to analog compounds like Y[VO_4_] and CdGa_2_S_4_.^58,59^ It is important to note that for wavelengths shorter than 650 nm it was not possible to have an SHG emission with sufficient signal-to-noise ratio. Indeed, for lower wavelengths, the second harmonic emission starts to be reabsorbed in the band gap (around 520 to 550 nm) and by the Urbach tale. A common trend is recognized in the SHG range (Figure S4). The SHG intensity is increased from 650 to 725 nm and decreased in the range of 850 to 950 nm. In the intermediate range between 725 and 850 nm, the values vary significantly for different measurement spots, not indicating a clear tendency. Materials that enable SHG at wavelengths higher than 800 nm have the potential to be used for any type of near-infrared applications, such as biological applications,^60^ solid-state laser technology,^61^ and SHG microscopy.^62^ To have a clearer view on the most suitable applications, more studies, like the efficiency, the (cyto)toxicity, or the SHG anisotropy, should be considered.

## 4 Conclusion

We present the syntheses of Na_3_[VS_4_] and K_3_[VS_4_] using a straightforward and scalable method. Both compounds are investigated using low-temperature SCXRD, PXRD, EDX, and solid-state NMR techniques. The X-ray results indicate the purity of both materials, while NMR measurements reveal small impurities (not detectable in the PXRD), as well as the existence of the [VS_4_] tetrahedra with slight deviations from the cubic symmetry. Dielectric studies indicate dielectric constant values of 76.52 and 103.67 for Na_3_[VS_4_] and K_3_[VS_4_], respectively. Ionic conductivity values are 2.41·10^–5^ and 1.36·10^–4^ mS·cm^–1^ at ambient temperature for Na_3_[VS_4_] and K_3_[VS_4_], respectively. UV–visible spectroscopy measurements determine the optical band gap energies around 2.1 eV for Na_3_[VS_4_] and around 2.3 eV for K_3_[VS_4_]. According to the noncentrosymmetric space group type of Na_3_[VS_4_], second harmonic generation is observed in a wide spectral region. The findings highlight new aspects of sulfido vanadate salts as multifunctional materials for potential optical and electrical applications.

## Supporting Information Available

Details of crystal structure refinement and crystal data, Rietveld structure refinement, EDX measurements and results, NMR measurements and results, and NLO measurements and results are provided in the ESI (PDF)
