# Supplementary material for: Optical and Electrical Properties of A3[VS4] (A = Na, K) Synthesized via a Straightforward and Scalable Solid-State Method
Source: Inorg Chem. 2024 May 31;63(24):11030–40. doi: 10.1021/acs.inorgchem.4c00551 (PMC11190978; doi:10.1021/acs.inorgchem.4c00551)
Supplement: Supplementary file 1 — ic4c00551_si_001.pdf [file ic4c00551_si_001.pdf]

Supplementary Information

Optical and Electrical Properties of $A_3[VS_4]$ (A = Na, K) Synthesized via a Straightforward and Scalable Solid-State Method

Mohammad R. Ghazanfari,[1] Laura Vittadello,[2, 3] Stephanie Bachmann,[4] Jakob Möbs,[5, 6] Rüdiger Bertermann,[7] Niklas Restel,[1] Felix Sauerwein,[2] Johannes C. Vrijmoed,[8] Johanna Heine,[5] Ann-Christin Pöppler,[4] Mirco Imlau,[2, 3] Günther Thiele[1,9,*]

1. Fachbereich Biologie, Chemie, Pharmazie, Freie Universität Berlin, Fabeckstr. 34-36, 14195 Berlin, Germany
2. Department of Mathematics/Informatics/Physics, University of Osnabrück, Barbarastraße 7, 49076 Osnabrück, Germany
3. Research Center for Cellular Nanoanalytics Osnabrück, Barbarastraße 11, 49076 Osnabrück, Germany
4. Institut für Organische Chemie, Universität Würzburg, Am Hubland, 97074 Würzburg, Germany
5. Department of Chemistry and Material Sciences Center, Philipps-Universität Marburg, Hans-Meerwein-Straße, 35043 Marburg, Germany
6. Department of Physics, University of Oxford, Parks Road, OX1 3PU Oxford, United Kingdom
7. Institut für Anorganische Chemie, Universität Würzburg, Am Hubland, 97074 Würzburg, Germany
8. Fachbereich Geowissenschaften, Freie Universität Berlin, Malteserstr. 74-100, 12249 Berlin, Germany
9. Institut für Anorganische und Analytische Chemie, Albert-Ludwigs-Universität Freiburg, Albertstr. 21, 79104 Freiburg, Germany

*Corresponding author: Dr. Günther Thiele; E-mail: guenther.thiele@fu-berlin.de

Contents

1. Details of crystal structure and single crystal XRD refinement
2. Details of PXRD measurements and Rietveld refinement
3. Details of EDX analysis
4. Details of solid-state NMR spectroscopic measurements
5. Tauc-Plots from diffuse reflectance UV-visible measurements
6. Details of NLO measurements and results
7. References

1. Details of crystal structure and single crystal XRD refinement

Crystal data: **1** (248.15 g·mol^{-1}) crystalizes in the tetragonal space group of $P\bar{4}2_1c$ (no. 114), a = b = 13.4403(6) Å, c = 7.9073(5) Å, V = 1428.39(16) Å^3, Z = 8, T = 130(2) K, ρ_{calc} = 2.308 g·cm^{-1}, $\mu(Mo\text{-}K_\alpha)$ = 2.619 mm^{-1}, *GooF* = 1.036, R_{int} = 0.0950, crystal dimensions (mm^3) = 0.361 × 0.156 × 0.112, 11321 reflections collected (4.286° < 2Θ < 54.926°). Multi-scan absorption correction was applied with *SADABS*.[S1] The final refinement values were R_1 = 0.0308 ($I > 2\,\sigma(I)$), and wR_2 = 0.0569 (all data).

Full data of the refinement details are reported in tables S1 to S3.

Table S1. Fractional atomic coordinates (×10^4) and equivalent isotropic displacement parameters (Å^2×10^3) for compound **1**. U_{eq} is defined as $\frac{1}{3}$ of the trace of the orthogonalised U_{IJ} tensor

Atom	*x*	*y*	*z*	U_{eq}
V5	2384.8(6)	4891.1(6)	2792.8(11)	8.24(19)
S3	3389.6(9)	4202.0(9)	1020.3(18)	11.0(3)
S6	1894.3(10)	6299.4(9)	1761.7(17)	12.5(3)
S8	1128.8(9)	3913.6(9)	3187.8(17)	11.7(3)
S2	3139.8(9)	5098.4(10)	5139.6(16)	12.6(3)
Na9	-450.4(14)	2557.7(15)	3535(3)	13.0(5)
Na4	2534.4(15)	3000.6(15)	5656(3)	16.6(5)
Na7	0	5000	736(4)	19.8(7)
Na1	5000	5000	2856(4)	18.3(6)

Table S2. Anisotropic displacement parameters (Å^2×10^3) for compound **1**. The anisotropic displacement factor exponent takes the form: $-2\pi^2[h^2a\cdot 2U_{11}+2hka\cdot b\cdot U_{12}+\ldots]$

Atom	U_{11}	U_{22}	U_{33}	U_{23}	U_{13}	U_{12}
V5	8.1(4)	8.2(4)	8.4(4)	0.0(3)	0.2(3)	0.3(4)
S3	10.6(6)	11.5(6)	11.0(7)	-1.0(5)	0.9(5)	2.3(5)
S6	15.4(6)	9.8(6)	12.2(7)	0.7(5)	0.8(6)	2.9(5)
S8	10.6(6)	12.5(6)	12.0(7)	1.5(5)	0.7(5)	-2.1(5)
S2	13.3(6)	14.7(6)	9.7(6)	-2.2(6)	-1.1(5)	-0.1(5)
Na9	13.2(10)	14.4(10)	11.3(11)	0.7(9)	-1.4(9)	-1.7(9)
Na4	16.8(11)	14.5(10)	18.3(12)	3.3(9)	-1.7(10)	1.0(9)
Na7	18.2(15)	28.3(17)	12.8(17)	0	0	6.8(14)
Na1	15.3(14)	18.7(15)	20.8(15)	0	0	-3.7(14)

Table S3. Bond lengths for compound **1** (Å)

Atom	Atom	Length		Atom	Atom	Length
V5	S3	2.1554(15)		S8	Na4	2.980(2)
V5	S6	2.1638(15)		S8	Na7^6	2.915(2)
V5	S8	2.1617(14)		S8	Na7	2.862(2)

V5	S2	2.1333(15)		S2	Na9[6]	2.883(2)
V5	Na9[1]	3.451(2)		S2	Na4[4]	3.248(2)
V5	Na4	3.409(2)		S2	Na4	2.963(2)
V5	Na7	3.5972(17)		S2	Na1[4]	2.963(2)
V5	Na1	3.5183(8)		S2	Na1	3.087(2)
S3	Na9[2]	2.854(2)		Na9	Na4[7]	3.487(3)
S3	Na9[1]	2.881(2)		Na9	Na4[1]	4.005(3)
S3	Na4[1]	3.004(2)		Na9	Na7	4.005(3)
S3	Na1	2.818(2)		Na9	Na7[6]	3.764(2)
S6	Na9[3]	2.845(2)		Na9	Na1[8]	3.660(2)
S6	Na9[1]	2.890(2)		Na4	Na4[6]	4.0823(10)
S6	Na4[4]	2.973(2)		Na4	Na4[1]	4.0824(10)
S6	Na4[5]	3.078(2)		Na7	Na7[1]	3.9536(3)
S6	Na7	3.1922(15)		Na7	Na7[6]	3.9537(2)
S8	Na9	2.811(2)		Na1	Na1[4]	3.391(6)
S8	Na4[1]	3.028(2)				

[1]1/2-Y,1/2-X,-1/2+Z; [2]1/2+X,1/2-Y,1/2-Z; [3]-X,1-Y,+Z; [4]+Y,1-X,1-Z; [5]1/2-X,1/2+Y,1/2-Z; [6]1/2-Y,1/2-X,1/2+Z; [7]-Y,+X,1-Z; [8]1/2-X,-1/2+Y,1/2-Z

Crystal data: **2** (296.48 g·mol^{-1}) crystalizes in the orthorhombic space group of *Pnma* (no. 62), a = 9.0902(12) Å, b = 10.4946(12) Å, c = 9.0832(11) Å, V = 866.52(18) Å^3, Z = 4, T = 130(2) K, ρ_{calc} = 2.273 g·cm^{-1}, $\mu(Mo\ K_\alpha)$ = 3.452 mm^{-1}, $GooF$ = 1.072, R_{int} = 0.0925, crystal dimensions (mm^3) = 0.227 × 0.124 × 0.117, 4918 reflections collected (5.932° < 2Θ < 52.734°). Multi-scan absorption correction was applied with *SADABS*.[S1] The final refinement values were R_1 = 0.0366 ($I > 2\ \sigma(I)$), and wR_2 = 0.0890 (all data).

Full data of the refinement details are reported in tables S4 to S6.

Table S4. Fractional atomic coordinates (×10^4) and equivalent isotropic displacement parameters (Å^2×10^3) for compound **2**. U_{eq} is defined as $\frac{1}{3}$ of the trace of the orthogonalised U_{IJ} tensor

Atom	x	y	z	U_{eq}
V2	7160.9(12)	7500	9840.0(12)	14.0(3)
K5	4494.6(12)	5449.1(10)	728.6(11)	18.5(3)
K6	6410.2(18)	7500	4128.2(18)	25.9(4)
S1	4973.4(18)	7500	10779.4(18)	16.7(4)
S3	8310.8(13)	9195.6(12)	10509.5(14)	20.5(3)
S4	6988.6(19)	7500	7489(2)	18.7(4)

**Table S5.** Anisotropic displacement parameters (Å^2×10^3) for compound **2**. The anisotropic displacement factor exponent takes the form: $-2\pi^2[h^2a{\cdot}2U_{11}+2hka{\cdot}b{\cdot}U_{12}+\ldots]$						
Atom	U_{11}	U_{22}	U_{33}	U_{23}	U_{13}	U_{12}
V2	11.9(6)	13.8(5)	16.2(6)	0	0.0(5)	0
K5	18.5(6)	16.7(5)	20.3(5)	0.7(5)	1.1(4)	-0.3(5)
K6	17.3(9)	34.7(10)	25.9(9)	0	-3.4(7)	0
S1	12.5(9)	19.3(9)	18.3(8)	0	1.3(7)	0
S3	16.2(7)	19.7(7)	20.6(8)	0	-0.2(7)	0
S4	17.2(9)	18.4(9)	25.7(7)	-4.5(5)	0.1(5)	-3.4(5)

**Table S6.** Bond lengths for compound **2** (Å)						
Atom	Atom	Length		Atom	Atom	Length
V2	K5	3.678(2)		K5	S1^4	3.3434(17)
V2	K5^1	3.934(2)		K5	S1	3.395(2)
V2	K5^2	3.934(2)		K5	S3^8	3.200(2)
V2	K5^3	3.9910(18)		K5	S3^9	3.326(2)
V2	K5^4	3.9910(18)		K5	S3^{10}	3.247(2)
V2	K5^5	3.678(2)		K5	S4^{11}	3.157(2)
V2	K6^1	3.974(3)		K5	S4	3.151(2)
V2	K6^6	3.954(3)		K6	S1^1	3.235(4)
V2	S1	2.164(3)		K6	S1^{12}	3.312(3)
V2	S3	2.150(2)		K6	S3^{11}	3.347(3)
V2	S3^5	2.150(2)		K6	S3^{13}	3.6970(19)
V2	S4	2.144(3)		K6	S3^9	3.347(3)
K5	K5^4	3.985(3)		K6	S3^{10}	3.6970(19)
K5	K6^7	3.7070(19)		K6	S4	3.097(3)

^{1}1/2-Y,1/2-X,-1/2+Z; ^{2}1/2+X,1/2-Y,1/2-Z; ^{3}-X,1-Y,+Z; ^{4}+Y,1-X,1-Z; ^{5}1/2-X,1/2+Y,1/2-Z; ^{6}1/2-Y,1/2-X,1/2+Z; ^{7}-Y,+X,1-Z; ^{8}1/2-X,-1/2+Y,1/2-Z

2. Details of PXRD measurements and Rietveld refinement

Rietveld structure refinement was conducted on the PXRD result of **1** and **2** to investigate the phase purity and obtain the structural properties of the powder. The structural parameters of the PXRD diffractogram including background, atomic temperature factor, preferred orientation, zero shift, micro strain, and crystallite size were refined using *GSAS II* software,[S2] according to the crystallographic information file (CIF) of the pure phases of **1** and **2** obtained from the single crystal measurement. The residual values including R_P, R_{WP}, and χ^2 of the refinement are 4.61%, 8.25%, and 1.99 for **1**, and 5.28%, 9.12%, and 1.96 for **2**, respectively.

3. Details of EDX analysis

To conduct the EDX measurements, two samples for each, **1** and **2**, were prepared under inert atmosphere and measured under high vacuum, however putting powder samples on the carbon sticks, and transferring to the device was carried out in room atmosphere, leading to the partial oxidization of the surfaces. Thus, to obtain the elemental stoichiometries, the oxygen and carbon impacts are omitted and the obtained values for sodium (potassium for **2**), vanadium, and sulfur were normalized accordingly. Point EDX measurements were performed for two different samples at selected locations on the sample as well as EDX maps over an area of the sample. Samples were transferred to a *Zeiss Sigma 300VP* field emission scanning electron microscope (FE-SEM), coupled with two *Bruker, Quantax Xflash 6*, 60 mm2, SSD, EDX detectors. For EDX measurements an optimal working distance of 8.5 mm, a beam aperture of 60 micrometer and a beam energy of 20 kV was used. The *Bruker Esprit 2.1* software was used to perform standardless EDX measurements and to quantify the data.

In the EDX spectrum of both **1** and **2** (Figure S1a and b), there are two additional peaks (overlapped with the intensity axis) for carbon at 0.277 eV, K_α and oxygen at 0.523 eV, K_α. In addition, there is a negligible peak at 1.74 eV, attributed to the noise due to the silicon detector. Figure S1 displays the results of the EDX measurements including the absorption pattern as well as the table of elemental ratios of **1** and **2**. According to the results, the measured ratio of Na:V:S and K:V:S are very close to the nominal stylometric ratio of 3:1:4, approving the acceptable purity of the compounds.

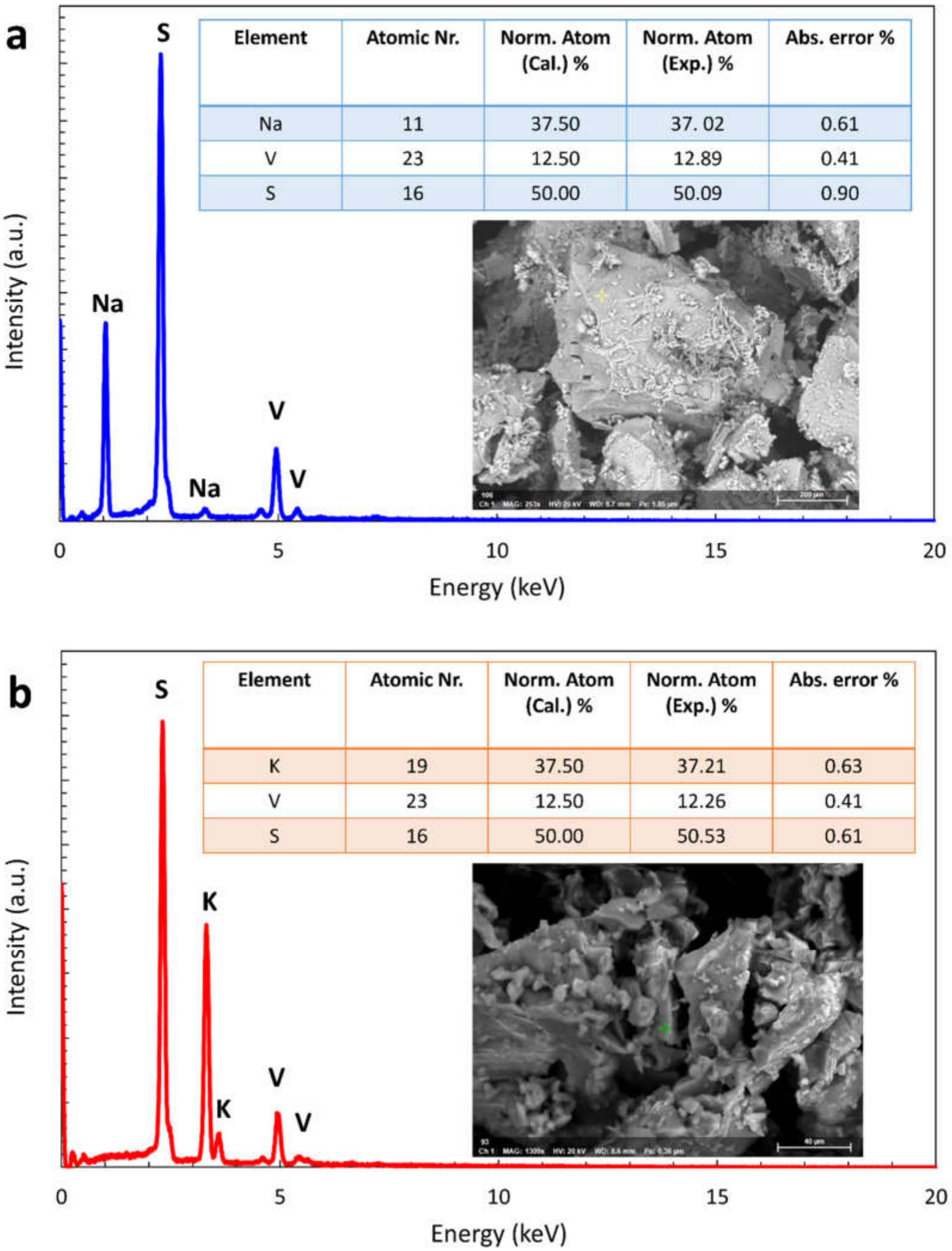

Figure S1. EDX spectrum of powder of (a) **1**, and (b) **2**, with the extracted amounts of the elemental ratio, and a micrograph of the powder from backscattered electron signals at an applied beam energy of 20 kV using scanning electron microscopy.

4. Details of solid-state NMR spectroscopic measurements

To identify the isotropic ^{51}V signals, the samples were measured at different spinning speeds.

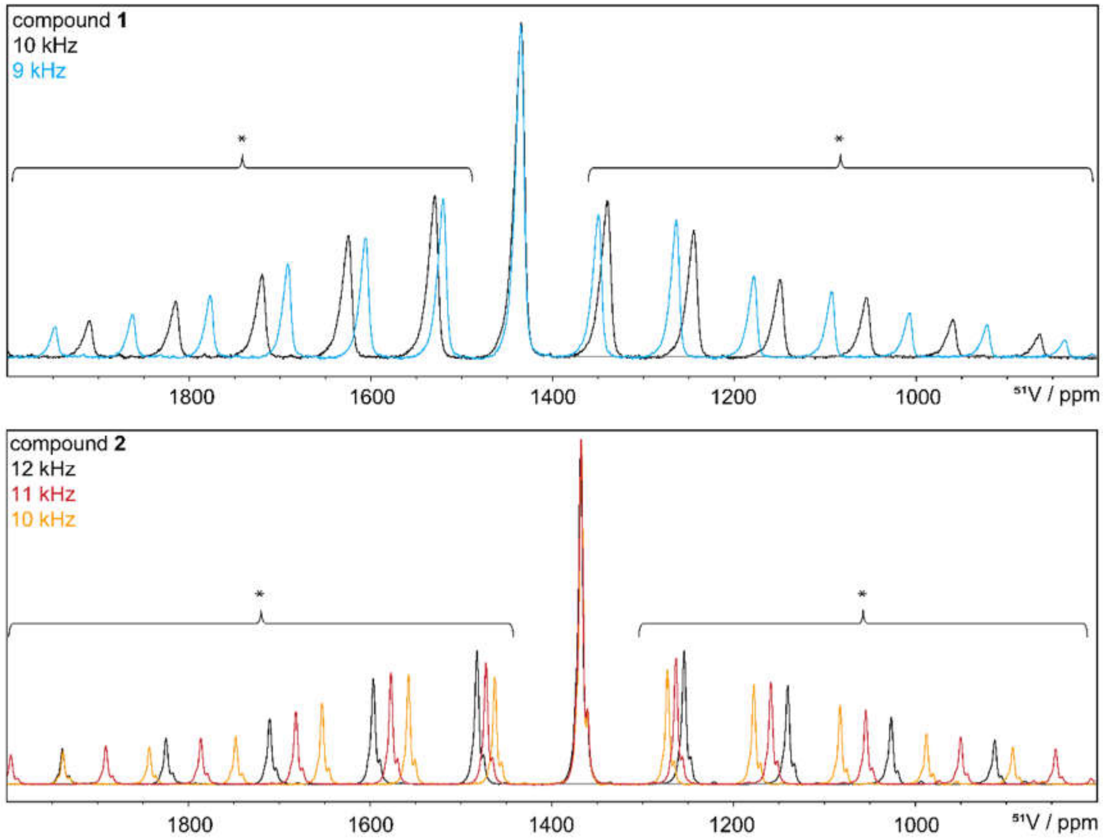

Figure S2. ^{51}V direct polarization (DP) solid-state NMR spectra of **1** and **2** at different spinning speeds. Spectra were measured with 16 and 32 scans, respectively, and a recycle delay of 60 s. Spinning side bands are marked by asterisks.

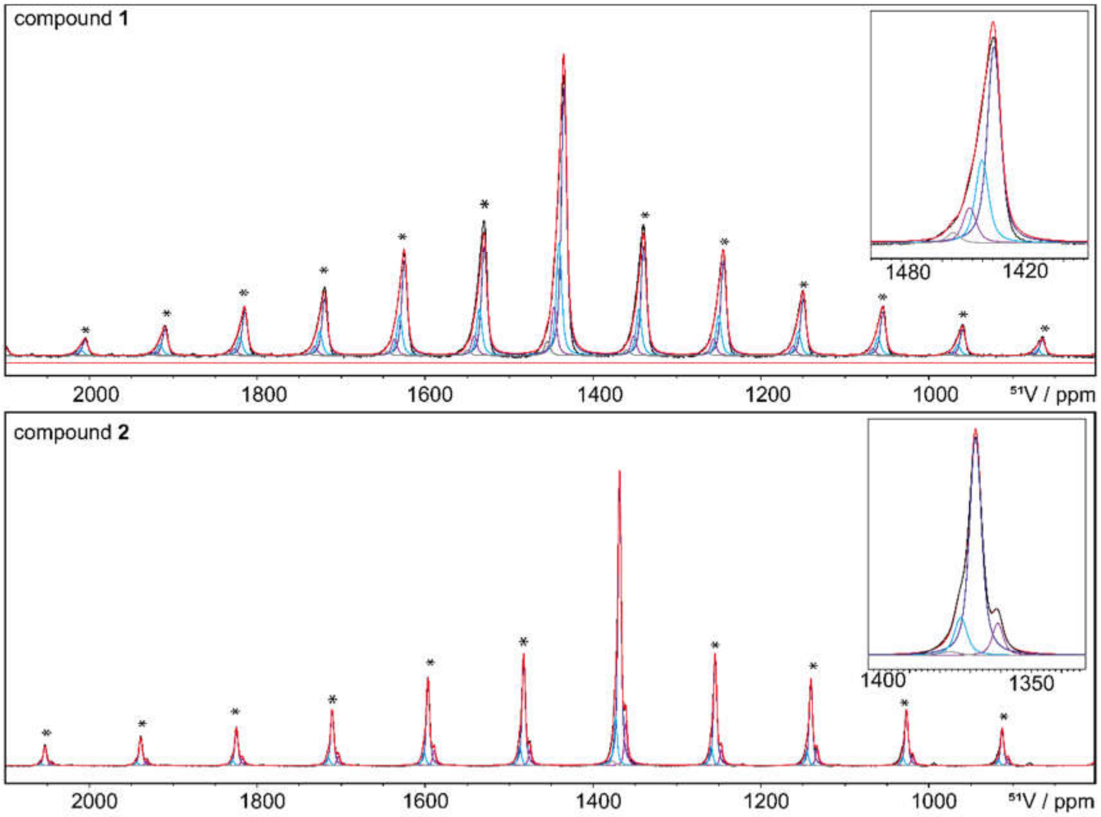

Figure S3. ^{51}V solid-state NMR spectra measured at 10 kHz and with 32 scans for **1** and 12 kHz and 16 scans for **2**. The isotropic signals and the signal pattern of **1** and **2** were fitted using the Solid Lineshape Analysis (sola) module version 2.2.4 (2013) of *Bruker TopSpin 4.3*. The experimental data (black) are displayed together with the envelope (red) of the individual fitted signals. Spinning side bands are marked by asterisks.

5. Tauc-Plots from diffuse reflectance UV-visible measurements

To determine the optical band gaps the raw data was transformed from reflectance R to absorption according to the Kubelka-Munk function

$$F(R) = \frac{(1-R)^2}{2R}$$

and then plotted as a Tauc-plot, where $(F(R) \cdot h\nu)^{1/n}$ is plotted against radiation energy. For a direct band gap n would be $\frac{1}{2}$, for an indirect band bap 2.[S3, S4] We assume a direct band gap for both compounds as the results were meaningless when choosing $n = 2$.

Note, that for the determination of the gap energies the absorption bands at lower energies were neglected because these are effects of distinct S-*p* to V-*d* charge transfer excitations and not part of the continuous bands.[S5, S6] Due to this overlap the given values should only been viewed as rough estimates.

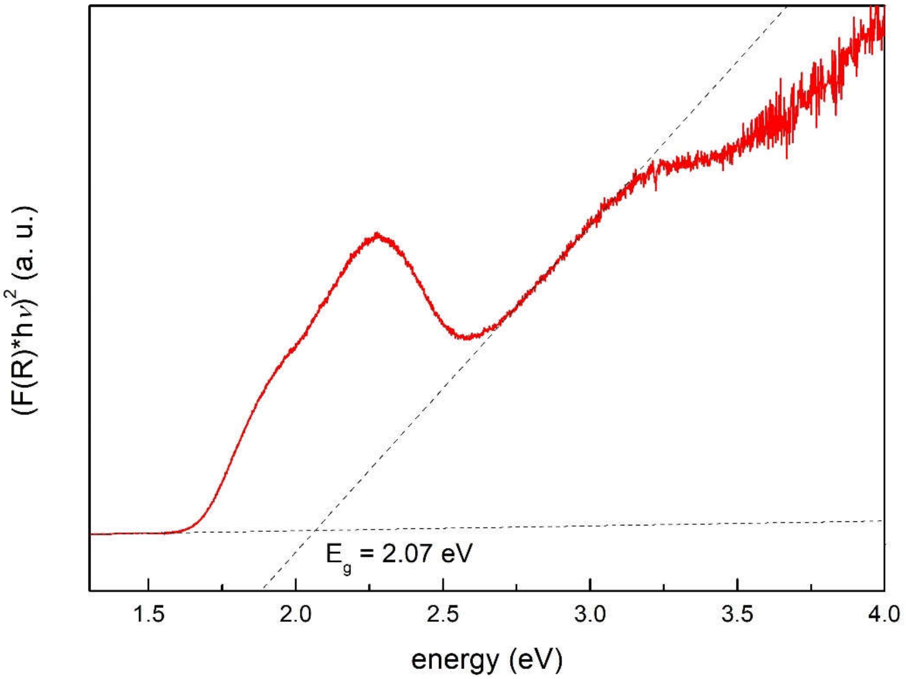

Figure S4. Tauc-Plot of **1**. The *x*-coordinate of the intersection of linear fits applied to the baseline and the absorption edge in the spectrum gives an estimation of the band gap energy.

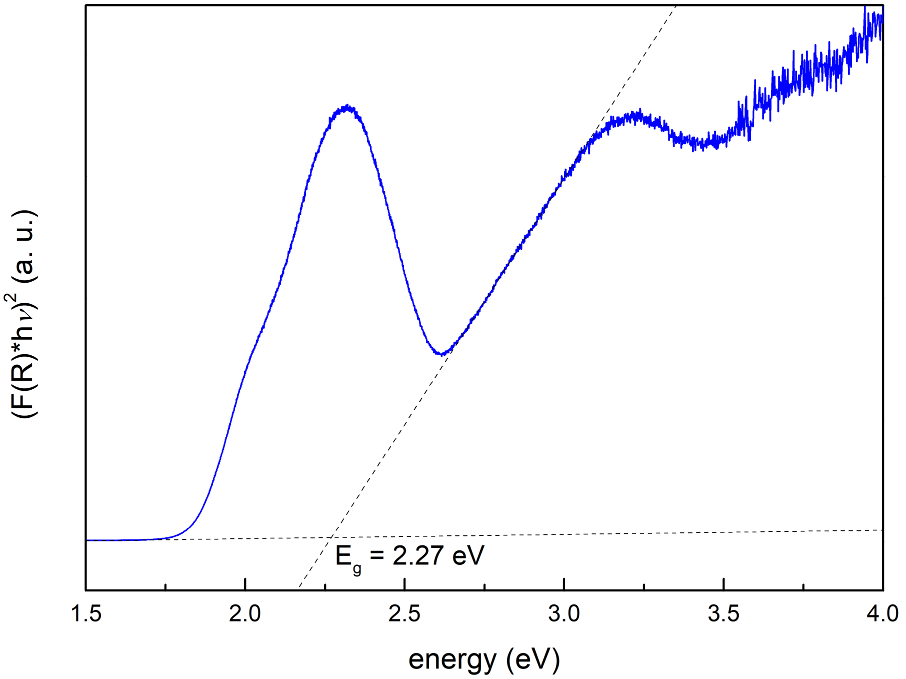

Figure S5. Tauc-Plot of **2**. The *x*-coordinate of the intersection of linear fits applied to the baseline and the absorption edge in the spectrum gives an estimation of the band gap energy.

6. Details of NLO measurements and results

In this study, the spectral efficiency of the SHG was measured. As the measurements described in the main text, the spectral efficiency of SHG is measured in reflection geometry. The average power of the laser, the repetition rate and the laser area were kept almost constant. Measurements in three different spots were acquired, whilst the results are presented in Figure S5. A common trend is recognized in the SHG range (Figure S5): The SHG intensity is increased from wavelengths of 650 nm to 725 nm and decreased in the range of 850 to 950 nm. In the intermediate range between 725 and 850 nm, the values vary significantly for different measurement spots, not indicating a clear tendency.

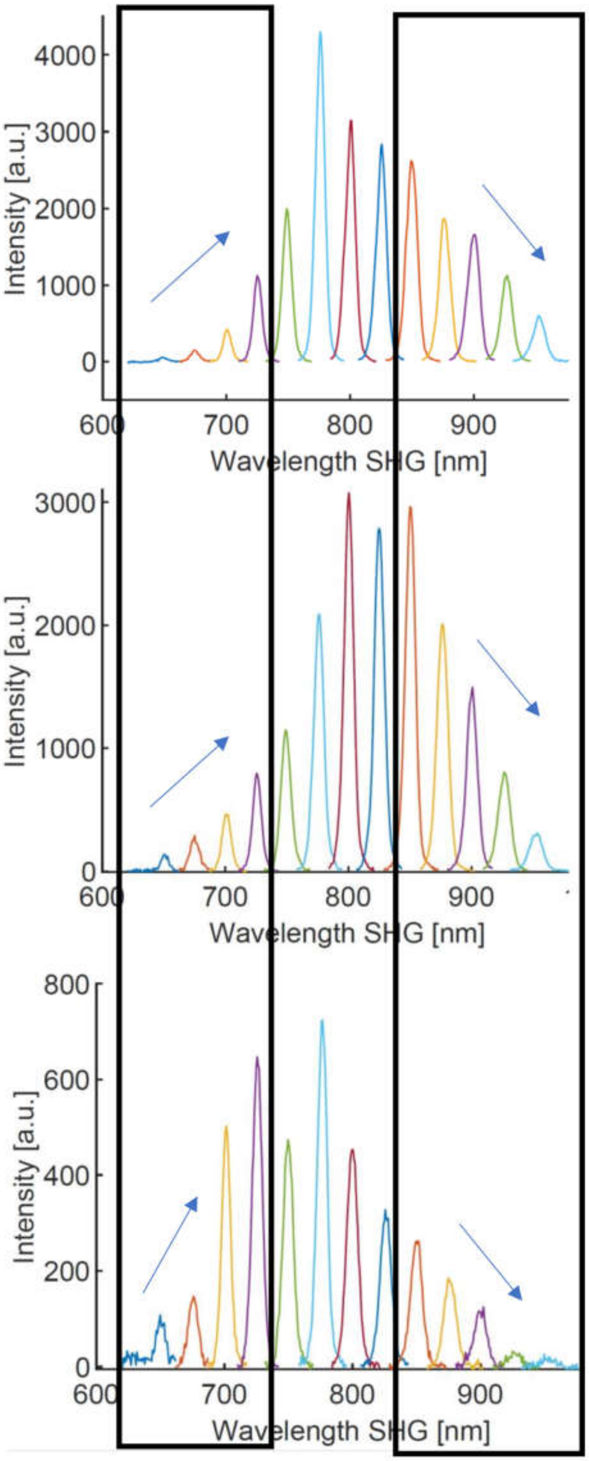

Figure S6. Spectral efficiency of the SHG measured in three different spots, indicating increasing and decreasing trends in the wavelength ranges of 650 to 725 nm, and 850 to 950 nm, respectively.

7. References

S1. Sheldrick, G. M. SADABS v. 2: Multi-Scan Absorption Correction, Bruker-AXS, WA, **2012**.

S2. Toby, B. H.; von Dreele, R. B. GSAS-II: The Genesis of a Modern Open-source All Purpose Crystallography Software Package. *J. Appl. Crystallogr.* **2013**, *46*, 544-549.

S3. Makuła, P.; Pacia, M.; Macyk, W. How to Correctly Determine the Band Gap Energy of Modified Semiconductor Photocatalysts based on UV-Vis Spectra. *J. Phys. Chem. Lett.* **2018**, *9*, 6814–6817.

S4. Michalow, K. A.; Logvinovich, D.; Weidenkaff, A.; Amberg, M.; Fortunato, G.; Heel, A.; Graule, T.; Rekas, M. Synthesis, Characterization and Electronic Structure of Nitrogen-Doped TiO_2 Nanopowder. *Catal. Today* **2009**, *144*, 7–12.

S5. Schnabel, S. Strukturchemie, elektronische Strukturen und spektroskopische Eigenschaften der Alkalimetallthiooxovanadate(V) $A_3VS_xO_{4-x}$. *PhD Thesis*, Albert-Ludwigs-Universität Freiburg, Freiburg, Germany, **2008**.

S6. Schnabel, S.; Röhr, C. Kalium-Thio/Oxo-Vanadate(V) $K_3[VS_xO_{4-x}]$ (x = 1–4) und $Na_3[VSO_3]$: Synthese, Strukturchemie, Eigenschaften. *Z. Naturforsch. B* **2008**, *63b*, 819-833.
